# Extracellular vesicles as nodal mediators of neuroHIV pathogenesis: a perspective

**DOI:** 10.20517/evcna.2026.123

**Published:** 2026-08-21

**Authors:** Satyajit Ghosh, Palsamy Periyasamy, Shilpa Buch

**Affiliations:** Department of Pharmacology and Experimental Neuroscience, University of Nebraska Medical Center, Omaha, NE 68198, United States.

## INTRODUCTION

HIV-associated neurocognitive disorders (HAND) remain a major clinical complication in people living with HIV (PLWH), affecting a substantial proportion of individuals despite the widespread use of combination antiretroviral therapy (cART)^[1]^. Approximately 30%-50% of PLWH continue to experience varying degrees of neurocognitive impairment, ranging from asymptomatic neurocognitive impairment to HIV-associated dementia^[2,3]^. Importantly, substance use disorder (SUD) is a common comorbidity in PLWH and has been recognized as an important modifier of HAND pathogenesis, as drugs of abuse can exacerbate neuroinflammation, disrupt blood-brain barrier **(**BBB) integrity, and potentiate HIV-mediated neuronal injury. Thus, although cART effectively controls peripheral viral replication, as PLWH continue to age, the combined effects of persistent viral proteins, substance use, and prolonged antiretroviral exposure result in sustained neurocognitive dysfunction and central nervous system injury. This shift has moved the focus away from direct viral toxicity alone toward intercellular communication pathways that amplify neuropathology, with particular emphasis on extracellular vesicles (EVs)^[4,5]^. EVs are now widely recognized as key mediators of central nervous system (CNS) signalling because they shuttle proteins, lipids, and nucleic acids among neurons, astrocytes, microglia, pericytes, and endothelial cells, thereby shaping neuroinflammation, synaptic integrity, and BBB function^[6,7]^.

In neuroHIV, EVs are especially important because they can package viral proteins, inflammatory mediators, and regulatory RNAs that reprogram recipient cell behaviour at a distance. Astrocyte-derived EVs (ADEVs), for example, may carry Tat-associated signals and pathogenic microRNAs that influence microglial activation, neuronal survival, and vascular homeostasis, while microglia-derived EVs (MDEVs) can further disseminate inflammatory signals that exacerbate neuronal injury. EVs also hold strong biomarker potential, as brain-derived vesicles detectable in cerebrospinal fluid (CSF) and blood have been linked to neurocognitive impairment as well as to markers of neuronal and glial injury in PLWH. Together, these findings position EVs as both mechanistic drivers and translational biomarkers of HAND, providing a useful framework for understanding viral persistence, CNS pathology, and disease processes in the era of cART.

This perspective integrates current evidence into a unified, EV-centered model of NeuroHIV pathogenesis. Within this framework, astrocyte-, microglia-, and neuron-derived EVs function as key intermediates that translate the convergent effects of persistent viral proteins, cART exposure, aging, and substance use into cell-type-specific responses across the neurovascular unit. HIV Tat- and opioid-focused EV pathways highlighted below represent well-characterized examples of how these diverse stressors converge on EV signalling to sustain CNS injury. Elucidating these EV-mediated circuits may reveal novel therapeutic targets and enhance biomarker discovery for HAND.

## EVs AND THE INDIRECT PATHWAY OF NEUROTOXICITY

### ADEVs in tat-mediated neuropathology

#### The Tat-miR-9-PTEN axis and microglial migration

HIV-1 Tat protein persists in the CNS even under suppressive cART and has been implicated in a wide range of neuropathogenic processes^[1]^. A foundational study demonstrated that exposure of human astrocytes to HIV-1 Tat induces the expression and selective enrichment of miR-9 in released EVs^[8]^. These Tat-stimulated ADEVs were subsequently internalized by microglia, leading to downregulation of the target phosphatase and tensin homolog (PTEN) through direct binding of miR-9 to the 3’ untranslated region (UTR) seed sequence of PTEN mRNA. This mechanism was confirmed *in vivo* and could be blocked by inhibiting exosome release or protecting the PTEN miR-9 binding site. Collectively, these findings established the Tat-ADEV-miR-9-PTEN axis as a seminal example of EV-mediated intercellular communication in neuroHIV, demonstrating that HIV-1 Tat can reprogram astrocytes to secrete EVs carrying functional miRNAs that modulate recipient microglial responses. However, independent validation of this specific signalling axis remains limited, and additional studies across diverse experimental models will be essential to determine its reproducibility, generalizability, and broader relevance to HIV-associated neuropathogenesis.

#### The Tat-miR-7-NLGN2 axis and synaptic impairment

Another study showed that a parallel pathway involving EV-mediated transfer of miR-7 from Tat-stimulated astrocytes to neurons affected synaptic architecture in recipient neurons. Specifically, exposure of astrocytes to HIV-1 Tat mediated the induction and release of EV-miR-7, which neurons took up, leading to downregulation of neuroligin 2 (NLGN2), a synaptic cell adhesion molecule critical for inhibitory synapse formation and function^[9]^. NLGN2 downregulation resulted in synaptic alterations that could be reversed by pretreatment of neurons with platelet-derived growth factor-CC (PDGF-CC), a neurotrophic factor. This finding not only identified EV-miR-7/NLGN2 as a direct mechanism of Tat-mediated synaptic injury but also highlighted PDGF-CC as a potential neuroprotective intervention.

#### Amyloid-carrying ADEVs and Alzheimer’s-like pathology

Previous work further extended the ADEV paradigm to include non-miRNA cargoes with direct neurotoxic potential. Importantly, this study showed that astrocytic HIF-1α (hypoxia-inducible factor 1 alpha) played a major role in HIV-1 Tat-mediated astrocytic amyloidosis, and that these toxic amyloids could be shuttled in ADEVs to the recipient neurons^[10]^. Tat-exposed astrocytes release EVs containing amyloids that induce neuronal amyloid accumulation, synaptic damage, and cognitive impairment. HIF-1α regulates both EV production and amyloid packaging, and silencing it reduces neuronal degeneration, behavioural deficits, and Alzheimer’s-like pathology, highlighting HIF-1α as a promising therapeutic target for HAND.

### MDEVs and NLRP3-mediated neuronal injury

#### The Tat-MDEV-NLRP3 axis

While astrocytes have been the primary focus of EV research in neuroHIV, microglia, the resident immune cells of the CNS, also release EVs with potent effects on neurons. HIV-1 Tat has been shown to upregulate the NLRP3 inflammasome pathway in microglia, activating these cells and regulating the release of MDEVs carrying significant levels of NLRP3 protein^[4,5]^. Tat-stimulated microglial EVs transfer NLRP3 to neurons, causing neuronal death, synaptodendritic damage, and impaired synaptic function. Knockdown of microglial NLRP3 reduces EV release and alleviates neuronal injury, identifying NLRP3 as a key mediator of EV-driven neurotoxicity.

This finding expands the role of NLRP3 beyond its well-characterized function in inflammation to include EV-mediated neurotoxicity, implicating it as a therapeutic target in HAND.

#### miR-21-TLR7 interaction and neurotoxicity

In another study, the miR-21-TLR7 axis represented a major conceptual advance in EV-mediated neuroHIV pathogenesis, showing that EV-miRs function not only as transported gene-regulatory cargo but also as extracellular immune ligands that directly activate neuronal innate immune receptors^[11]^. In simian immunodeficiency virus (SIV) encephalitis, brain-derived EVs enriched with miR-21 promote neuronal injury through sequence-specific activation of TLR7, triggering receptor-interacting serine/threonine-protein kinase 1 (RIPK1)-dependent necroptosis rather than apoptosis. This study identifies EV-packaged miR-21 as a pathogenic danger signal that links macrophage/microglial inflammation to innate immune-mediated neurodegeneration, establishing EV-miRNAs as active drivers of neuroHIV pathology rather than merely biomarkers.

### Opioid-EV interplay and exacerbation of neuroHIV pathology

#### Morphine-miR-29b/PDGF-B axis and neuronal viability

Opioid abuse is a major comorbidity in PLWH and has been shown to accelerate HAND progression. A seminal study demonstrated that astrocytes exposed to both morphine and HIV-1 Tat release EVs enriched in miR-29b^[12]^. Neurons took up these EVs, resulting in decreased expression of the target gene, PDGF-B (platelet-derived growth factor B), an important neurotrophic factor and direct target of miR-29b. Treatment of SH-SY5Y cells with exosomes from morphine/Tat-treated astrocytes resulted in decreased neuronal viability, establishing EV-miR-29b as a mediator of opioid-enhanced neurotoxicity in neuroHIV. Consistent with this mechanism, increased expression of miR-29b with a concomitantly reduced expression of PDGF-B was observed in the basal ganglia of morphine-dependent SIV-infected macaques compared with SIV-infected controls.

#### Morphine-miR-23a/pericyte/BBB axis

A structurally parallel yet anatomically distinct EV axis centres on pericytes and the BBB. Morphine-stimulated human primary astrocytes demonstrated release of the ADEVs enriched in miR-23a, and these levels were found to be time-dependently upregulated following morphine exposure. miR-23a expression was also found to be elevated in the basal ganglia of morphine-dependent macaques^[13]^. Morphine-ADEVs, when exposed to pericytes, were found to robustly induce pericyte migration. Direct morphine exposure of pericytes to morphine failed to recapitulate this effect, clearly implicating EV cargo rather than receptor-level opioid signalling on pericytes themselves. Mechanistically, miR-23a in morphine-ADEVs was transferred from astrocytes to pericytes, where it downregulated the target PTEN, in turn activating Akt signalling, ultimately culminating in pericyte migration and increased monocyte influx. Furthermore, heterogeneous nuclear ribonucleoprotein complex A2/B1 (hnRNP A2/B1) was identified as a sorting factor for miR-23a into the EVs. Silencing hnRNP A2/B1 selectively blocked miR-23a enrichment in ADEVs without affecting its intracellular expression. *In vivo*, morphine reduced pericyte coverage of brain microvessels and increased monocyte infiltration, linking EV-associated miR-23a to BBB disruption and neurovascular dysfunction. Together, these findings implicate the hnRNP A2/B1-miR-23a pathway in opioid-induced CNS injury. However, like the Tat-miR-9-PTEN pathway, evidence supporting this mechanism has thus far been derived largely from a single research group, underscoring the need for independent validation across diverse experimental models and clinical settings.

#### Morphine-miR-138/TLR7 axis in microglial activation

Another opioid-EV pathway involves EV-mediated pattern recognition receptor signalling in microglia. Morphine-stimulated astrocytes released ADEVs enriched in miR-138, which, when taken up by microglia, induced robust microglial activation mediated by engagement of the GUUGUGU motif of miR-138 with the microglial endosomal TLR7^[14]^. This EV-miRNA-TLR7 signalling cascade introduced a noncanonical function of EV-miRNAs, akin to damage-associated molecular pattern (DAMP)-like signals that directly stimulate innate immune receptors, rather than acting solely through classical mRNA target repression. *In vivo* validation demonstrated that morphine administration increased microglial activation in the thalamus of wild-type mice, whereas TLR7-deficient mice failed to exhibit this activation despite morphine exposure, underscoring TLR7 as an essential sensor for miR-138-bearing ADEVs.

## EVs AND THE DIRECT PATHWAY OF NEUROTOXICITY

HIV-1 viral proteins, including Tat, gp120, and Nef, contribute directly to neurotoxicity and neuronal injury, and some of these proteins can also be packaged into EVs/exosomes^[15]^. Tat has been detected in exosomes released by astrocytes, supporting a role for EV-mediated Tat transfer in neuroHIV pathogenesis^[16]^. Nef, a 27-35 kDa accessory protein, has been implicated in viral persistence and neuroimmune dysfunction and can promote neuronal injury through mechanisms involving inflammatory signalling and cytoskeletal remodelling^[17,18]^. Several studies have shown that glial cells can package Nef into EVs, although the efficiency appears to depend on the cell type. In one study, Nef-GFP transfection of microglia, astrocytes, and SH-SY5Y cells demonstrated that only microglia released Nef-containing exosomes, and that these vesicles disrupted BBB integrity^[17]^. In contrast, another study demonstrated that primary human astrocytes expressing Nef released Nef-containing EVs, and these ADEVs were taken up by neurons, where they induced oxidative stress, neuronal dysfunction, axonal and dendritic degeneration, increased tau expression, and impaired action potential firing^[18]^.

More broadly, these findings suggest that EV-associated viral proteins could amplify neuroinflammation and neurodegeneration in HIV infection and contribute to HAND pathogenesis.

## NEURONAL-DERIVED EVs AS PERIPHERAL BIOMARKERS

### Brain-to-blood EV transfer in HIV

Beyond their role as mediators of local CNS pathology, EVs may serve as accessible biomarkers reflecting CNS status in peripheral biofluids^[19]^. Investigation of EVs in HIV-1 transgenic (Tg) rats revealed that neuronal-derived EVs are enriched in both brain and serum, suggesting that long-term exposure to HIV-1 viral proteins enhances the release of neuronal EVs and their transfer to the systemic compartment.

Advances in EV characterization have established their potential as biomarkers for HAND. Elevated cerebrospinal fluid EV levels and proteomic enrichment of proteins linked to glial activation, neuroinflammation, synaptic dysfunction, cellular stress, and BBB impairment closely reflect ongoing CNS pathology in individuals with HAND^[20]^. Similarly, plasma-derived neuronal EVs have shown promise as peripheral biomarkers of HIV-related cognitive impairment. In studies using L1 cell adhesion molecule (L1CAM)-based enrichment of neuronal EVs, individuals with neuropsychological impairment exhibited higher levels of neuronal injury-associated proteins, including high mobility group box 1 (HMGB1), neurofilament light, and amyloid beta, compared with neuropsychologically normal individuals. These findings thus suggest that neuronal EV cargo likely reflects ongoing neurodegeneration in the brain^[21]^. EVs may also be useful in studies of comorbid substance use. In one investigation, plasma L1CAM-positive EVs were elevated in cigarette smokers, although HIV infection alone did not significantly alter the EV population; astrocyte-associated EVs marked by glial fibrillary acidic protein (GFAP) were elevated in PLWH with a history of alcohol use^[22]^. In addition, findings from the HIV Tg rat model indicate that chronic exposure to HIV viral proteins is associated with increased L1CAM-positive EVs in both the brain extracellular space and plasma, suggesting that this model could be useful for biomarker discovery and mechanistic studies^[19]^. In PLWH, plasma-derived neuronal EVs containing elevated levels of HMGB1, neurofilament light chain (NfL), and amyloid-β have been associated with poorer neuropsychological performance, suggesting that these cargoes reflect ongoing neuronal injury and neuroinflammation^[20-22]^. Similarly, cerebrospinal fluid-derived EVs from individuals with HAND are enriched in proteins associated with synaptic dysfunction, glial activation, inflammatory signalling, and BBB disruption, underscoring their potential as biomarkers of disease severity.

A major challenge is determining whether EV biomarkers are specific to HAND, as many candidates, including NfL, HMGB1, amyloid-β, and inflammatory EV cargoes, are also altered in other neurodegenerative and neuroinflammatory disorders. Therefore, multimarker EV signatures integrating neuronal, astrocytic, and microglial cargoes with HIV-related clinical parameters may provide greater diagnostic accuracy. Clinical translation is further hindered by the lack of standardized EV isolation, enrichment, and cargo quantification methods. Future studies should adopt harmonized workflows, normalize EV-associated RNA and protein levels to EV particle number and validated EV markers, and follow MISEV guidelines to improve reproducibility and clinical applicability.

Overall, EVs, particularly brain-derived and neuronal EVs, represent promising minimally invasive biomarkers for tracking HIV-associated brain injury, neuroinflammation, and HAND progression.

## THERAPEUTIC OPPORTUNITIES

Therapeutic strategies emerging from these studies centre on targeting EV biogenesis, cargo sorting, miRNA signalling, neurotrophic support, and inflammatory pathways. Inhibition of EV release, for example, with GW4869, has been shown to reduce Tat-induced EV-miR-9 transfer and attenuate microglial migration, highlighting EV secretion machinery as an upstream intervention point. Disrupting selective cargo loading, such as the hnRNP A2/B1-miR-23a interaction in astrocytes, could prevent miR-23a enrichment in ADEVs and protect pericytes without broadly impairing miRNA function. Modulation of HIF-1α further reduces EV biogenesis and amyloid cargo, thereby alleviating synaptodegeneration. Complementing these approaches, miRNA-based therapeutics offer additional precision: target protectors that shield PTEN from miR-9 or miR-23a can restore normal cellular function, while anti-miR-138 strategies may limit microglial activation through TLR7, particularly when delivered via engineered EVs. Engineered EV platforms have also shown promise; for example, miR-124 delivery reduced drug-induced microglial activation, supporting their potential as therapeutic carriers^[23]^. Neurotrophic factor supplementation, including PDGF-CC and PDGF-BB^[24]^, has been shown to rescue neurodegeneration and reverse synaptic deficits, reinforcing their neuroprotective potential. Targeting inflammatory pathways such as NLRP3 and TLR7 represents a promising therapeutic strategy for NeuroHIV, as their inhibition reduces synaptodendritic injury and EV-mediated microglial activation. However, major translational challenges remain. Broad inhibition of EV biogenesis using agents such as GW4869 may disrupt physiological EV functions critical for CNS homeostasis, immune regulation, and tissue repair. Likewise, effective, cell-specific delivery of miRNA therapeutics, neurotrophic factors, and engineered EVs across the BBB remains difficult. Most supporting evidence is derived from *in vitro* or animal studies, underscoring the need for validation in clinically relevant NeuroHIV models incorporating chronic HIV protein exposure, antiretroviral therapy, aging, and substance-use comorbidities. Accordingly, future therapeutic development should prioritize the precise modulation of pathogenic EV signalling rather than global inhibition of EV biogenesis.

## FUTURE DIRECTIONS

Collectively [Figure 1], these studies support an integrated EV-centered model of NeuroHIV pathogenesis involving three interconnected circuits. In the Tat-ADEV-microglia circuit, HIV-1 Tat stimulates astrocytes to release EV-miR-9 and EV-miR-7. EV-miR-9 promotes microglial migration through PTEN downregulation, whereas EV-miR-7 disrupts neuronal synaptic integrity by suppressing NLGN2. ADEVs also transfer amyloid cargo via HIF-1α-dependent pathways, contributing to Alzheimer’s-like synaptodendritic injury. In the Tat-MDEV-neuron circuit, Tat-activated microglia release NLRP3-enriched EVs that promote neuronal death and synaptic damage. In the opioid-ADEV circuit, morphine enhances hnRNP A2/B1-mediated EV release, enriching miR-23a, miR-138, and miR-29b, which impair pericyte function and BBB integrity, activate microglia through TLR7, and reduce neuronal viability by suppressing PDGF-B, respectively.

**Figure 1:**
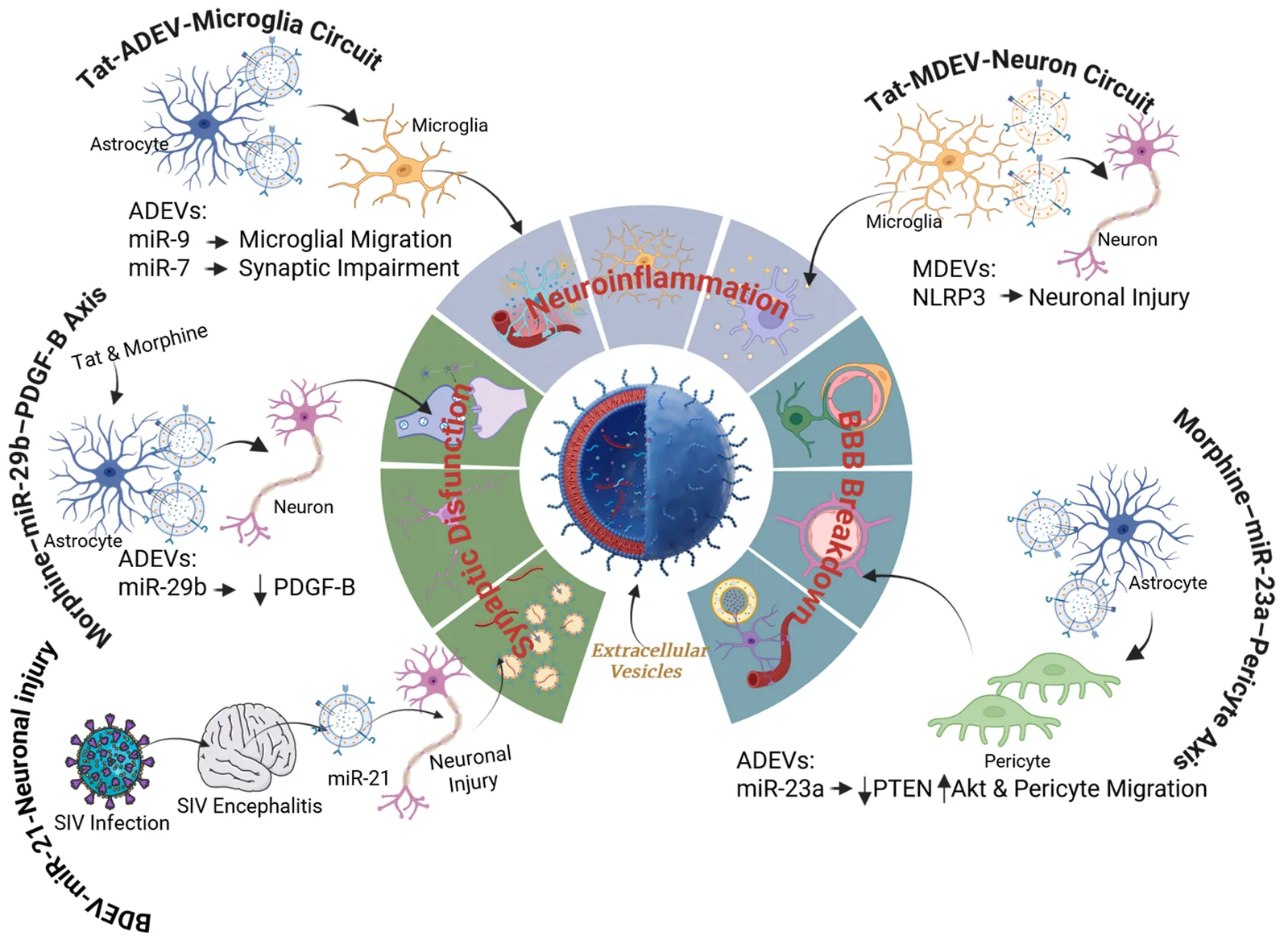
Schematic illustration of EV-mediated signalling pathways that contribute to HAND in the setting of persistent HIV-1 viral proteins and opioid exposure. HIV-1 Tat stimulates astrocytes to release ADEVs enriched with pathogenic microRNAs and toxic cargo. EV-associated miR-9 is transferred to microglia, where it suppresses PTEN, promoting microglial migration and activation. Tat-induced ADEVs also deliver miR-7 to neurons, resulting in downregulation of NLGN2, impaired synaptic integrity, and neuronal dysfunction. HIV-1 Tat also activates microglia and the NLRP3 inflammasome, resulting in the release of MDEVs containing NLRP3. Transfer of these EVs to neurons amplifies neuroinflammation, induces synaptodendritic degeneration, and promotes neuronal cell death. BDEV-associated miR-21, released during lentiviral CNS infection, activates neuronal TLR7, triggering RIPK1-dependent necroptosis and further contributing to neurodegeneration. Opioid exposure further exacerbates EV-mediated neuropathology. Morphine stimulates astrocytes to release ADEVs enriched in miR-23a and miR-29b. Transfer of miR-23a to pericytes suppresses PTEN, activates the Akt signalling pathway, promotes pericyte migration and loss of vascular coverage, and increases blood-brain barrier permeability, facilitating peripheral monocyte infiltration into the CNS. EV-associated miR-29b suppresses neuronal PDGF-B, reducing neuronal viability and increasing susceptibility to injury. EV: Extracellular vesicle; HAND: HIV-associated neurocognitive disorders; ADEV: astrocyte-derived extracellular vesicle; miR-9: microRNA-9; PTEN: phosphatase and tensin homolog; miR-7: microRNA-7; NLGN2: neuroligin-2; MDEV: microglia-derived extracellular vesicle; NLRP3: NLR family pyrin domain containing 3; BDEV: brain-derived extracellular vesicle; miR-21: microRNA-21; TLR7: Toll-like receptor 7; RIPK1: receptor-interacting serine/threonine-protein kinase 1; miR-23a: microRNA-23a; Akt: protein kinase B; miR-29b: microRNA-29b; PDGF-B: platelet-derived growth factor B; CNS: central nervous system.

Despite these advances, important challenges remain. Most studies rely on bulk EV isolation and conventional markers, limiting the ability to distinguish exosomes from other EV subtypes or assign vesicles to specific CNS cell populations. Future work should integrate orthogonal isolation methods, single-vesicle and spatial profiling, cell-specific EV labelling, and standardized reporting to define cargo-specific mechanisms. Independent validation of key signalling pathways, including the Tat-miR-9-PTEN and morphine-miR-23a axes, across cellular, animal, and human models is also needed.

Clinical translation requires validation of EV-associated miRNAs, including miR-9, miR-7, miR-23a, miR-138, and miR-29b, as biomarkers in cerebrospinal fluid and blood, with correlations to HAND severity, viral suppression, cART exposure, opioid use, and cognitive decline. Future experimental models should better reflect the complexity of NeuroHIV by incorporating viral proteins, antiretroviral therapy, aging, and substance-use comorbidities while defining EV communication within the neurovascular unit. Therapeutic strategies should prioritize selective targeting of pathogenic EV populations or cargo rather than global inhibition of EV biogenesis to preserve physiological CNS communication and improve long-term safety.

## CONCLUSION

EVs are emerging as central mediators of NeuroHIV pathogenesis, linking viral persistence, glial activation, substance-use comorbidity, neurovascular dysfunction, and neuronal injury. Rather than passive byproducts of cellular stress, EVs actively transmit pathogenic signals across the neurovascular unit. Astrocyte-, microglia-, and disease-associated brain-derived EVs transport regulatory miRNAs, inflammatory proteins, viral stress signals, and amyloid cargo that reprogram recipient cells through pathways involving PTEN, NLGN2, TLR7, NLRP3, and PDGF-B. Key EV-associated miRNAs, including miR-9, miR-7, miR-23a, miR-138, miR-29b, and simian immunodeficiency virus encephalitis (SIVE)-associated miR-21, regulate microglial migration, synaptic integrity, BBB function, innate immune activation, and neuronal survival. Opioid exposure further amplifies these effects by enhancing astrocytic μ-opioid receptor signalling, hnRNP A2/B1-dependent miRNA sorting, and EV-mediated disruption of neuronal and vascular homeostasis. This EV-centered framework shifts the understanding of NeuroHIV from isolated cellular events to interconnected networks of intercellular communication and injury propagation. To achieve clinical translation, future studies must resolve EV heterogeneity, define CNS cell-specific EV populations, validate disease-associated cargo signatures in well-characterized patient cohorts, and identify pathogenic EV subsets and therapeutically actionable cargoes. These advances will be essential for developing reliable biomarkers and targeted interventions to mitigate persistent neuroinflammation and neuronal dysfunction despite effective antiretroviral therapy.

## DECLARATIONS

### Copyright

© The Author(s) 2026.
